# Examining Sustainability Factors for Organizations that Adopted Stanford’s Chronic Disease Self-Management Program

**DOI:** 10.3389/fpubh.2014.00140

**Published:** 2015-04-27

**Authors:** Michiyo Tomioka, Kathryn L. Braun

**Affiliations:** ^1^Office of Public Health Studies, University of Hawai‘i at Mānoa, Honolulu, HI, USA

**Keywords:** chronic disease, health promotion, evidence-based, minority groups, sustainability

## Abstract

In 2006, funds were received to replicate Stanford’s Chronic Disease Self-Management Program (CDSMP) among eldercare providers in Honolulu. This case study, conducted 1 year after the close of the initial 3-year replication grant, explored factors for sustaining the delivery of CDSMP, with an aim to create guidelines for cultivating sustainability. Face-to-face semi-structured interviews were conducted with one representative from each of eight eldercare agencies, with the representative specified by the agency. Representatives discussed the presence and strength (low, medium, or high) of sustainability factors, including readiness, champions, technical assistance, perceived fit of CDSMP with their agency, CDSMP modifiability, perceived benefits of CDSMP, and other. Only three of the eight agencies (38%) were still offering CDSMP by the end of 2010. Agencies who sustained CDSMP rated higher on all sustainability factors compared to those that did not sustain the program. Additional factors identified by representatives as important were funding and ongoing access to pools of elders from which to recruit program participants. When replicating evidence-based programs, sustainability factors must be consciously nurtured. For example, readiness must be cultivated, multiple champions must be developed, agencies must be helped to modify the program to best fit their clientele, evaluation findings demonstrating program benefit should be shared, and linkages to funding may be needed.

## Introduction

Demand for preventing, delaying the onset, and managing chronic diseases has escalated. Attention is being given to expanding replication of evidence-based health promotion programs, those proven to work, to address chronic disease (1). Several federal agencies recommend that service providers adopt evidence-based health promotion programs rather than “reinvent the wheel” in efforts to help older adults maintain health and independent living for as long as possible (2, 3). Yet, studies on how organizations learn about, adapt, and sustain such programs are limited (4, 5).

In replicating an evidence-based program, organizations need adequate knowledge and skills in adapting the program to fit local circumstances while maintaining fidelity and evaluating the program to assure that it achieves the outcomes promised in the original research (6–8). Much of the extant literature outlines the many challenges of translating scientific knowledge to community practice (9–13). These include: (1) resistance to new practice modalities; (2) lack of organizational buy-in; (3) lack of specific goals and standards in translating the evidence; and (4) rigidity of evidence-based practice that cannot be molded to meet specific needs of the applied setting or target population (4, 6, 14).

These adaptation barriers also influence the long-term continuation of evidence-based programs, and more attention is being focused on ways to assure widespread availability of evidence-based programs (15–18). Useful models such as RE-AIM (Reach, Effectiveness, Adoption, Implementation, and Maintenance) help guide replication processes and evaluation (19).

At the same time, researchers have summarized and advanced definitions of program sustainability, identified factors associated with sustainability, and developed conceptual frameworks to understand program sustainability (15, 17, 20–22). According to literature review of sustainability research by Wiltsey-Stirman et al. (18), one of the most cited definitions of sustainability evolved from work of Scheirer (15) and Shediac-Rizhallah and Bone (17) and is: “the integration of the new program into ongoing organizational systems.”

Scheirer’s framework for program sustainability posits four phases of program adoption: (1) initiation; (2) implementation; (3) level of use (full or partial); and (4) sustainability (sustained, discontinued, or replaced) (15). Based on her literature review, agencies that sustain new programs likely agree that the program can be modified to fit their organization, see the program as fitting their organization’s mission, perceive program benefits, have champions for the program, and have access to technical assistance while adopting the program.

The purpose of this study was to describe and determine the important factors that supported or hindered sustainability of the Stanford’s Chronic Disease Self-Management Program (CDSMP) among eldercare service providers in Hawai‘i. CDSMP was developed to empower people with various chronic diseases to take control of their health (23). Participants attend six 2.5-h sessions (one per week). Facilitators share knowledge and use motivational interviewing techniques to engage participants, who make weekly action plans to help themselves take small steps toward changing a behavior of their choice. Numerous studies of CDSMP have shown that people who participate in this program feel better, have better control over the symptoms of their chronic diseases, and are better able to talk to their physicians (23, 24). Although the original test of CDSMP was conducted with Caucasians (25), it has been successfully adapted to fit Hawai‘i’s multicultural communities (26).

The implementation of CDSMP in Hawai‘i was supported by Hawai‘i Healthy Aging Partnership (HHAP), formed in 2003 to increase access to health promotion programs among Hawai‘i older adults with chronic conditions. HHAP members include professionals from government offices for aging and public health, elder care agencies, and the university. The process of CDSMP adaptation began in August 2006 when HHAP was awarded a 3-year grant from the Administration on Aging (AoA). HHAP members developed CDSMP implementation and evaluation plans, assessed readiness to implement CDSMP, and coordinated training for CDSMP leaders. Implementation in two of Hawai‘i’s four counties began in July 2007. It was expanded statewide in 2008 with funds from the National Council on Aging (NCOA). The sustainability phase began in 2009, when the original implementation funding ended. The purpose of this study, guided by Scheirer’s sustainability framework, was to better understand the process and factors associated with sustainability of CDSMP in Hawai‘i.

## Materials and Methods

### Design, settings, participants

Although CDSMP was implemented statewide, the findings reported here were gathered as a part of the Honolulu case study, which was conducted to understand the process of organizational change for CDSMP adoption. In the Honolulu case study, data from state and county government, community organizations, and older adults were examined to investigate “how” and “why” organizations in Honolulu adapted, implemented, and sustained CDSMP successfully or unsuccessfully (27). The Honolulu case study was approved by University of Hawai‘i Institutional Review Board. This paper reports on a portion of data collected, specifically from the eight service providers in Honolulu that started to replicate CDSMP in 2007.

For the Honolulu case study, we identified three phases of the CDSMP adoption path. The first 6 months of 2007 was considered as the Initiation Phase, when HHAP began planning replication and training agency personnel in CDSMP delivery. The Delivery Phase ran from June 2007 to June 2009, during which time staff members from multiple agencies were trained and then delivered CDSMP to older adults and participated in ongoing fidelity monitoring and evaluation. The Sustainability Phase began in July 2009, when original funding ended. This paper reports on the Sustainability Phase of new-program adoption.

The eight providers included multi-purpose social service organizations (designated as A and D), community health centers (designated as B, F, G, and H), a community college (designated as E), and a community meals program (designated as C). Four providers (A, B, C, and D) had a closer relationship with the Honolulu County Area Agency on Aging than the other four (E, F, G, and H), because they had been funded by the Honolulu County Area Agency on Aging for other programs. Five providers (A, B, C, E, and G) were involved with HHAP during the statewide planning of CDSMP adoption, whereas three providers (D, F, and H) joined HHAP when CDSMP training was held. Each provider chose the employee to be interviewed in this study.

### Measures

The investigators developed an interview guide that asked about the five sustainability factors identified by Scheirer (15): (1) champions; (2) technical assistance; (3) perceived fit of the program; (4) program modifiability; and (5) perceived benefits of the program. A sixth factor, readiness to replicate, was added, as it was considered important to HHAP partners. After discussing each factor, participants were asked to rate its importance to sustainability as low, medium, or high. Finally, they are asked to identify other sustainability factors (Table 1).

**Table 1:** **Summary of sustainability factors assessed**.

Sustainability factors	Sample questions
1. Readiness	Describe your “readiness” to replicate CDSMP. For example, how adequate was training in the program, data collection, and program monitoring forms? How prepared was your agency?
2. Champions^a^	Describe your experience with program champions for CDSMP? Who and how many people from your organization were helping with CDSMP, and in what ways? What did these champions do? Comment on their effectiveness.
3. Technical assistance^a^	How does your organization have access to technical assistance to sustain the program? Comment on the availability and usefulness of technical assistance as you replicated CDSMP.
4. Program-organization fit^a^	How does CDSMP match your organization’s culture or mission? Comment on the level of “fit” between CDSMP and your agency.
5. Program modifiability^a^	Describe your ability to change or modify CDSMP that fit your clients and your agency. Describe your experience making program modifications while trying to maintain fidelity to the original CDSMP design.
6. Perceived program benefits^a^	How did organizational leaders and worker feel CDSMP impacted your clients? How do you think CDSMP benefited the people you served? In what ways has your involvement in CDSMP benefited clients, staff, and your organization?
7. Other (open-ended)	How do you think CDSMP will be sustained by your agency? What are the major factors that contributed to long-term sustainability?

*^a^Identified by Scheirer (15)*.

Readiness refers to an individual’s and agency’s sense of preparedness to replicate the program. A champion is an agency employee who plays a key role in adapting, delivering, and/or sustaining CDSMP in the agency. Technical assistance refers to help employees could access when they had questions or encountered problems in CDSMP implementation and sustainment. Perceived fit of the program implies a similarity between the new intervention and the parent organization’s mission and culture. Program modifiability refers to the level of satisfaction that the agency has with the modifications that it can make to the evidence-based program (e.g., to better fit its clientele and agency structure) without jeopardizing the behavior-changing components of the program. Perceived benefits of the program include feelings of staff and clients (which may or may not be based on evaluation data) that the program is making a positive impact.

These factors were identified by Scheirer (15), with the exception of readiness (item 1). Readiness as a sustainability factor was identified through our review of the sustainability literature and discussion with funders, who considered organizational readiness an important first step in adoption of CDSMP.

Although providers continue to offer CDSMP, data for this study of sustainability factors were collected in late 2010. At that time, the first author (MT) conducted face-to-face, semi-structured interviews with the eight community provider representatives in Honolulu. The interview questions were sent to the representatives ahead of the interview to help them prepare. All eight representatives provided a written consent. Interviews were held at the representatives’ offices and took about an hour. Six individuals allowed their interviews to be audio taped and, for two, hand notes were made. All interviews were transcribed into text files.

### Analysis

Ratings of low, medium, or high were noted for each respondent for each sustainability factor. The discussion of each factor and the discussion of other possible factors were read independently by two researchers. For the most part, discussion of *a priori* sustainability factors served to give examples of, expand on, and contextualize each factor, which was useful in understanding its ranking (low, medium, or high). Analysis of responses to the open-ended question about other possible factors required the two researchers to discern themes in the data and then code responses into themes. These were discussed in a meeting, and differences were resolved by re-reading the interview transcript together and having further discussion until consensus was reached. There were no major disagreements during the analysis process.

## Results

**Figure 1:**
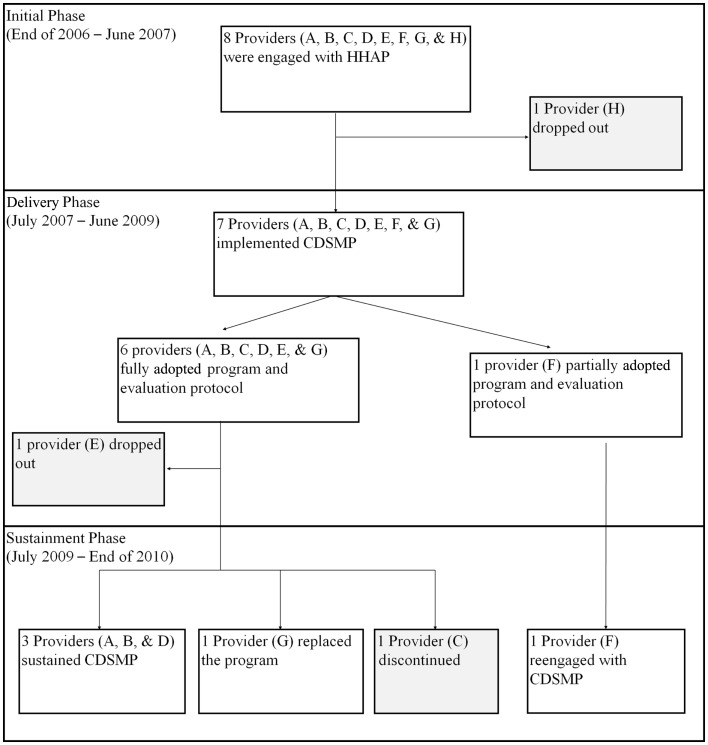
**Providers progress over the 4 years**.

The progress of the eight providers who volunteered to replicate CDSMP in Honolulu is shown in Figure 1. One provider (H) dropped out during the Initial Phase (the first 6 months) because of the organization decided that it could not dedicate staff time to deliver CDSMP. Thus, only seven of the eight organizations entered the 2-year Delivery Phase. Two providers (E and F) dropped out before the end of that phase. For Provider E, two staff members completed CDSMP training and offered it twice in the community, but the organization felt that it was too time consuming to recruit and track clients and chose to discontinue. Provider F was not able to fully implement the program with its fidelity monitoring and evaluation requirements. Of the five entering the Sustainment Phase in mid-2009, only three providers (A, B, D) were still sustaining CDSMP in late 2010. Provider G had replaced CDSMP with another program, and Provider C discontinued the program. Provider F decided to reengage with CDSMP at this time.

During the analysis process, it became clear that organizations that sustained CDSMP had more supports throughout the process than the organizations that were unable to sustain CDSMP. Table 2 shows a summary of rating results from the interview.

**Table 2:** **Summary of interview rating results**.

Outcome	Org	Readiness	Champions				Program- organization fit	Program modifiability	Perceived benefits	Technical assistance
			No. of prog champ	Effectiveness	Supervisor support	Participants champ				
Sustained	A	H	2	H	H	Yes	H	H	H	H
	B	H	3	H	H	Yes	H	H	H	H
	D	H	2	H	H	Yes	H	H	H	H
Replaced	G	H	1	H	H	?	H	H	H	H
Discontinued	C	M	0	L	L	No	L	H	H	H
Reengaged	F	L	1	M	L	No	L	H	M	H
Dropped out in implementation phase	E	L	0	L	M	No	L	M	M	M
Dropped out in initial phase	H	L	0	L	L	No	L	M	–	M

*H, high; M, medium; L, low; ?, unknown*.

### Readiness

Thinking back to the Delivery Phase, providers described and rated their level of readiness to replicate CDSMP. The five providers (63%) who scored their organization as “high” had noted in their discussion of readiness that their staff had spent time learning about the concept of evidence-based programing prior to program adoption, felt well trained in CDSMP and data collection, were motivated to pilot CDSMP in their community, had identified potential CDSMP participants, had established policies and procedures related to CDSMP, and had purchased a CDSMP license. The three providers who scored medium or low remembered some uncertainty within their organization and perhaps some miscommunication with HHAP as to the costs associated with CDSMP licensing, the coordination of CDSMP workshops, and the need to participate in fidelity monitoring and evaluation.

### Champions

Respondents agreed that having champions was very important to sustainability, and the organizational representatives that reported high champion effectiveness were most likely to be from organizations that sustained CDSMP. The transcript-analysis process, however, distinguished three types of champions, including program champions, participant champions, and supervisor champions.

Respondents defined a program champion as someone who had been trained to lead CDSMP, had a passion for it, was very committed, was able to promote it, and had the drive to expand it. Although all organizations had staff trained in CDSMP, not all could identify a program champion at the time of the interview, while some agencies reported as many as three program champions. The three sustained programs reported having more than one program champion at their organization.

Interview findings suggested that the most successful program champions had relatively flexible schedules, which allowed them to offer CDSMP during or outside of work hours, and their job descriptions included CDSMP. They had strong skills in teamwork and took time to educate other branches of the organization in CDSMP to increase organizational buy-in. They advocated for other staff in the agency to attend CDSMP leader training. They networked with CDSMP leaders at other agencies, which helped them find a substitute or second workshop leader when needed. They also were seen as role models by program participants and provided support to CDSMP leaders at other organizations. As one provider noted: “*Both champions have been enthusiastic and* …* try to promote, try to get more funding, try to get more people, are involved* …* and they wish they were able to do more*.”

The two providers who reported no program champion might have had a program champion initially, but this person changed jobs or became too busy to lead and advocate for CDSMP. One provider said: “*We don’t have a champion because we had to take care of other things*…*Champion requires that one person constantly pushes CDSMP*.” Sustained organizations reported that they would be able to continue to sustain the program as long as they had leaders and trainers on board. To facilitate this, HHAP continues to provide CDSMP leader trainings and encourage organizations to continuously send new staff or volunteers to training so that they could keep enough leaders within their organizations.

A participant champion was described as someone who had graduated the CDSMP (attending four or more sessions out of six), realized benefits from the program, and was willing to share their story about CDSMP. By talking about the benefits they received, they helped to recruit other participants for the program. Many providers felt that the word-of-mouth strategy was the most effective approach to attract new participants.

A supervisor champion was described as a manager who supported the delivery of CDSMP within the organization and supported the program champions in leading CDSMP sessions. Supervisor champions always sent an agency representative to the statewide HHAP meetings and saw CDSMP as a valuable service to offer agency clients. Interview analysis suggested that all sustained agencies had a supervisor champions. Respondents for the other four agencies felt that their supervisors were not strong champions. Two of them said that their supervisors oversaw a variety of projects and remained relatively uninvolved with CDSMP replication efforts. The other two providers reported that they did not get any support from their supervisors because their supervisors had unrealistic expectations of CDSMP, for example, that it would take less time than it did, would be more modifiable that it was, or would generate revenue. The discrepancy between the expectations of these supervisors and those of the staff trained in CDSMP discouraged staff from championing the program in their agencies.

### Program-organization fit

Five providers (63%) reported that CDSMP fit well with their organizational mission and goals. They valued the program’s concept of empowerment and its goal to help clients improve health and maintain independence through self-management. They also felt that their participants would appreciate the program’s motivational interviewing approach.

Two providers rated program-organizational fit as medium. They felt that CDSMP fit their general mission – to improve the well-being of older adults – but not the type of service they provided, in this case meal delivery and college courses.

### Program modifiability

Five out of eight providers (63%) reported that the modifications of CDSMP by HHAP (with permission of Stanford) to fit Hawai‘i’s multicultural population helped them attract enrollees. This was done by including local examples and expressions of local culture in the curriculum (26). One provider gave this example: “… *our participants are not fluent English speakers* …* so it takes double the time to explain things* …*. We serve local food that they like or fit with their culture, they feel happy even though they work hard during the session. We also offer certificate of completion with leis.a little recognition for their hard work and they were very happy about it*.” Providers who reported high program modifiability also spent time to develop local marketing tools that included pictures of local older adults and symbols that resonated with Hawai‘i’s cultures. The three providers who rated medium or low in program modifiability noted disappointment that the program materials were not available in the languages of their target groups (e.g., the various Pacific Islander languages, like Samoan, Chuukese, and Marshallese) and that the program required two leaders to deliver each workshop. They also reported that the structured outline and scripted format of the workshop were too foreign for their clients.

### Perceived benefits

One agency was not able to rate this factor because it dropped out prior to implementing CDSMP. Of the seven remaining providers, five rated CDSMP benefit to clients as “high” and perceived CDSMP was a good investment. They reported seeing improvements in the health of their clients and related success stories of participants who had lost weight, were exercising more, and/or were keeping better track of their health. One provider noted: “*I see them really change and keep hearing their stories* … *We had a quite a few that really struggled with making action plans. Boy, when they get it, they get it and they got so excited, you know, the first time they come back, they were so proud that they got something accomplished. I just think* …* you know, to me, it’s really had a big impact on people’s lives*.” They also saw benefits for their staff, many of whom had incorporated CDSMP tools (e.g., problem solving and developing action plan) into their daily activities and had gained confidence in public speaking. One provider used some of the CDSMP tools for staff training. They also mentioned the benefit of receiving evaluation results from HHAP specific to their agency to share with supervisors and funders. Hearing good stories from their participants and seeing the positive evaluation data further boosted their confidence in replicating CDSMP. The two providers who were rating this factor as “medium” noted that some of the agency’s staff and clients were unable to grasp or apply CSDMP self-management strategies.

### Technical assistance

Six of the eight providers (63%) reported that the technical assistance that they received from HHAP was very useful. Technical assistance was provided through HHAP’s monthly meetings, from the evaluation team, and from Stanford. At monthly meetings, providers were able to exchange ideas on how to leverage resources, to recruit and retain participants and leaders, and to carry-out the evaluation protocol. Meeting attendance also helped providers to develop strong skills in working with other providers. These relationships were useful when they needed to find a substitute leader for a CDSMP session or borrow books and other program materials for a workshop. A provider said: “*We are fortunate to have partners for CDSMP. I can call [Agency name], if I have questions. They also helped us and clarify things for us. One time, we did not have CDSMP books, and I called them* …* It was very helpful*. “Some providers reported that the Stanford website and email listserv were useful and helped motivate them to continue offering CDSMP. The providers who rated technical assistance as medium indicated that they wanted more support to recruit participants and more clarification of program requirements.

### Other sustainability factors

Interviewees were asked to identify other factors related to sustainability. Two were identified – having access to potential participants (four providers) and having access to additional funding (seven providers).

Because most of the providers were offering CDSMP to their existing clientele, they initially did not encounter problems finding participants. However, at some point they had provided CDSMP to all willing clientele. For the most part, agency clients were willing to participate in the program, and attendance was high (26), but “*once we went through all of the participants, then we did not have any more new participants* …* we do not have a large turnover in our clients, so we could not expand our numbers.”* Provider B noted that their organization was established to serve a specific community. After all willing clients completed CDSMP, their organization had to consider the advantages and disadvantages to enrolling people from other communities. Provider A felt that the CDSMP was important enough to continue because it fit so well with their program. This provider’s solution was to conduct CDSMP workshop with a combination of participants who have done it before and any new participants that they could find.

Additional funding was also a critical factor to sustain CDSMP. One provider stated “*in the long-term, always funding is needed, things cost money and staff time, plus money for licensing [and] when the new books come out* …* all of those things cost something.”* Although most of the providers were able to access additional funding through the HHAP’s awarded grants, sustained organizations’ interviewees also wrote proposals to other funders to support CDSMP. They also used cost-saving strategies, such as holding workshops at no-cost sites and creating a library of workshop materials that could be loaned to (rather than purchased for or by) participants.

## Discussion

Evidence-based health promotion programs are developed in research settings, and replicating them in real-world settings can be challenging (4, 5). This study examined factors related to sustainability of CDSMP by Honolulu County providers a year after initial funding ended. Depending on presence and strength of these factors, providers varied in their sustainment of CDSMP as shown in Figure 1.

As Scheirer (15) proposed, new-program sustainability can be enhanced by having many champions (and several types of champions), ensuring program fit with the organizational mission, allowing some modifications to the program so it can better fit clientele, seeing benefits of the program, and having access to technical assistance. In addition to these five factors, this study identified three more factors that appear to contribute to sustainability of CDSMP – readiness, access to additional funding, and access to potential participants.

Readiness can be cultivated by providing training about evidence-based programing, fidelity monitoring, and program evaluation, along with training in the intervention to be adopted (13). For non-profit organizations, external funding to support added programs is essential, especially in light of cutback to social services and the piloting of the “reimbursement-for-service” model by many eldercare service providers. Although continued funding was not cited explicitly by the majority of studies of program sustainability reviewed by Scheirer (15), she noted that many of these programs had in fact found alternative funding to maintain new programs.

Exhausting potential clients can occur in agencies that work within small geographic areas, have a fixed number of clients that they are allowed to serve, and/or have low client turnover (28). This is especially true in Hawai‘i, where providers receiving funds through the AAAs are contracted to serve a specified number elders, often in a defined target area. With low turnover in clientele, all willing participants can participate in CDSMP over the course of several years. Also, some service providers in Hawai‘i serve elders who speak languages for which CDSMP is not available. It may be that CDSMP is more sustainable in Hawai‘i’s health maintenance organizations that serve thousands of clients, as a portion of their clients would likely be diagnosed with chronic disease each year. Meanwhile, HHAP members have expressed a desire to learn about and replicate other evidence-based programs that could benefit their clientele. Already, a number of providers in the state are replicating EnhanceFitness with good success (29).

This study explored CDSMP sustainability among eight eldercare providers in one of Hawai‘i’s four counties, and only one representative from each organization was interviewed. Although organizations selected to be interviewed the individual most closely involved in CDSMP adaptation, the interviewee may not represent the whole organization.

Also, because the interview asked about sustainability after initial funding ended, the results may have been compromised by inability to recall events, especially for those organizations that discontinued CDSMP, and by social desirability bias. Future examination of new-program sustainability would benefit from prospective study and inclusion of multiple representatives of an organization.

Despite the limitations, this study was able to confirm the importance of the sustainability factors proposed by Scheirer (15), and added three more, which may be specific to the Hawai‘i context of CDSMP replication. The clear message from this study is that planning for sustainability should start before replicating evidence-based programs. It requires tremendous effort to translate evidence-based programs, to build provider capacity, to implement a new program (or new practice) in real-world setting, and to sustain it. These findings can help guide healthcare workers and organizations to plan and sustain the adoption of evidence-based programs.

## Conflict of Interest Statement

The authors declare that the research was conducted in the absence of any commercial or financial relationships that could be construed as a potential conflict of interest.

This paper is included in the Research Topic, “Evidence-Based Programming for Older Adults.” This Research Topic received partial funding from multiple government and private organizations/agencies; however, the views, findings, and conclusions in these articles are those of the authors and do not necessarily represent the official position of these organizations/agencies. All papers published in the Research Topic received peer review from members of the Frontiers in Public Health (Public Health Education and Promotion section) panel of Review Editors. Because this Research Topic represents work closely associated with a nationwide evidence-based movement in the US, many of the authors and/or Review Editors may have worked together previously in some fashion. Review Editors were purposively selected based on their expertise with evaluation and/or evidence-based programming for older adults. Review Editors were independent of named authors on any given article published in this volume.
